# Supplementary material for: Social predictors of the transition from anomie to deviance in adolescence
Source: PLoS One. 2022 Jun 22;17(6):e0269236. doi: 10.1371/journal.pone.0269236 (PMC9216585; doi:10.1371/journal.pone.0269236)
Supplement: Attachment — Submitted filename: _Response to Reviewers.docx [file pone.0269236.s001.docx]

Dear Editor and Reviewers,

Thank you very much for your feedback, recommendations and support for improving our paper, *Social predictors of the transition from anomie to deviance in adolescence*. We totally agree with your observations and we did our best to solve all the issues. We hope the new re-elaborated form is more adequate to the journal standards.

Please find bellow our answers to each comment from editor and reviewers.

Answers to Editor:

**1. Please ensure that your manuscript meets PLOS ONE's style requirements, including those for file naming**

We have made the following corrections/upgrades:

- included the line numbers

- double-spaced the paragraphs

- used the correct file name (Manuscript)

**2. Please improve statistical reporting and refer to p-values as "p<.001" instead of "p=.000".**

*The p-values are reporting as “p<0.001”*

We have had .000 only in two tables and we have changed it with <0.001, hope this is acceptable.

**3. We note that the grant information you provided in the ‘Funding Information’ and ‘Financial Disclosure’ sections do not match. When you resubmit, please ensure that you provide the correct grant numbers for the awards you received for your study in the ‘Funding Information’ section.**

We have received funding only for the publishing fee, not for the research.

**4. In your Data Availability statement, you have not specified where the minimal data set underlying the results described in your manuscript can be found. PLOS defines a study's minimal data set as the underlying data used to reach the conclusions drawn in the manuscript and any additional data required to replicate the reported study findings in their entirety. All PLOS journals require that the minimal data set be made fully available**

Unfortunately, it is a legal restriction to provide the free access of the data. We have to keep the initial explanation, all the data belong to Planet Youth Iceland: *due to internal and ethical rules and regulations, the whole data set is not to be made available for public sharing/ open access; however, the reasonable requests for data will be analysed and other interested researchers may receive data like: the values behind the means, standard deviations and other measures reported; the values used to build graphs, labels etc.*

**5. Please include your full ethics statement in the ‘Methods’ section of your manuscript file. In your statement, please include the full name of the IRB or ethics committee who approved or waived your study, as well as whether or not you obtained informed written or verbal consent. If consent was waived for your study, please include this information in your statement as well.**

It is included from line 173 to line 185

**Answers to Reviewer 1:**

Dear reviewer,

Thank you very much for your comments and critics, it has helped us very much to understand the weakness of our article and to increase its scientific value.

**1. It is not clear in the abstract the statistical analyzes that were carried out and what was presented as the objective of the study ("identify socio-cultural factors that may operate predictors of alienation and of deviant or even delinquent behavior"), is not aligned with what was presented as the main result ("Bucharest young people do not exhibit deviant behaviors or only to a minor degree, and that this is largely determined by a positive environment in terms of social relations, economic status, and level of culture.") . Furthermore, what is presented as the main result in the abstract is not congruent with the data presented in the manuscript, since 20% of the sample presented a type of behavior classified as “delinquency” and 12% as “high deviance” (Table 8).**

We have rewritten the abstract in accordance with this observation.

**2. In the introduction, the concepts of anomie and alienation are well defined and with a presentation of previous literature. Despite this, other variables that are addressed in the article (the risk factors assessed, such as parental monitoring, peer support, substance abuse, etc.) are not discussed based on the literature.**

We have extended the introduction by including the other variables used in the analysis.

**3. The method would require a characterization of the participants (age and gender) and a presentation of the psychometric parameters of the instrument adopted. In addition, more detail on the statistical analysis used would be necessary, describing the complete analysis process (data processing, choice of variables, descriptive statistics performed, procedure for carrying out logistic regression). As there is no description of the analysis procedure, it is not possible to know and to assess the rigor used in the process.**

We have included the socio-demographic profile of the sample and we have remade the statistics, in a clearer way: two dependent variables, anomie and deviant behaviour, with several independent variables from various socio-cultural factors included in the survey. We hope it is more adequate now.

**4. In the results, there is a presentation of definitions of scale variables and their composition that should be presented as “measures” in the method. There is no alignment between what is proposed to analyze the method and the analyzes presented in the results.**

Indeed, we have moved these scales (and two other new ones: the level of anomie and time spent on leisure activities on the Internet) to the Method section. We have adjusted the research objective to the results. We have rewritten almost completely the Results section.

**5. In the discussion, there is not enough discussion with literature data about the results found and the conclusions are not aligned with the results presented.**

We have extended the discussion and we have included the relevant literature data.

**ANSWERS TO REVIEW 2**

First of all, thank you very much for your so friendly review and so empathic approach. Please receive our deep gratitude for this!

**1) I suggest that the title, “Transition from anomie to delinquency in adolescence: Profiles and associated factors”, be reconsidered, because the study does not exactly reflect the profile of young people, but rather the factors that associate the transition from anomie to delinquency.**

Indeed, we have reformulated the title to be more appropriate to the study main idea. The new title is: *Social predictors of the transition from anomie to deviance in adolescence. Case study: the 2018 Youth Planet research in Bucharest, Romania*

**2) In the Introduction, the authors refer to some studies that correlate the predictors of what could potentially trigger a transition from anomie to delinquency; however, none of them discuss the social or structural conditions that potentiate such situation. In developing countries, for instance, macroeconomic conditions prevail over individual and even community conditions regarding the experience of unruly and deviant adolescence. I believe it is important to emphasize this comparative aspect, which would strengthen the study and allow its scope to be expanded beyond subjective conditions and personal inabilities to comply or not with societal rules.**

We have included in the Introduction some aspects related to juvenile delinquency in Romania.

**3) This study also sought to respond to the following question: “What are the most relevant factors associated with juvenile delinquency in Bucharest, Romania?” To this end, the authors resourced to a 17-item questionnaire. To better contextualize the research, I suggest that the authors include some information, if available, on the state of the art of juvenile delinquency in Romania and, in particular, in Bucharest, because only the data presented do not show whether delinquency is high or low among young Romanians.**

We have included in the Conclusions section some statistics related to the juvenile delinquency in Romania.

**4) The Methodology does not clearly present the profile of the study participants: there is need to explain, for example, the profile of the young people who responded to the questionnaire: age group, sex and gender identity, social class, ethnicity, in order to better understand the eligibility criteria of the study.**

We have included the socio-demographic profile of the sample.

**5) The Results and their statistical analysis faithfully demonstrate and answer the research question.**

We have almost completely remade the statistics and rewritten the Results section accordingly.

**6) Regarding the Discussion, there is need for a more comprehensive analysis of both the results found and the findings of the literature that support the arguments. How does the theory of anomie, proposed by Merton (1968), assists us with thinking about the transition from anomie to delinquency in young Romanians? What pre-existing conditions do these young people have that motivate them to go beyond culturally and socially determined limits? Making good use of the studies and theories presented here could better support the evidence of the results.**

We have made some adjustment of the Discussion section in accordance with the results from the upgraded statistics; we know that there are still some aspects that can be improved and, if you consider it necessary, we can go deeper with the theoretical contextualisation.

**7) In addition, the authors state that the amount of time spent on communication and information technologies (CIT) by today's youth emerges as a predictor of the high level of deviant behavior, but not necessarily of delinquent acts. To support this argument, it is important to review communication theories on the excessive and abusive use of CTI among young people.**

We have detailed the negative consequences of the abusive use of NICT (especially of accessing the online universe from one’s personal mobile phone without adult monitoring).

Thank you very much for your support!

On behalf of the authors,

Bogdan Nadolu

bogdan.nadolu@e-uvt.ro

Timisoara, Romania, April 09, 2022
